# The role of interleukin-17 in neurological disorders

**DOI:** 10.1080/19768354.2025.2510994

**Published:** 2025-06-03

**Authors:** Seung Eun Park, Ana Flávia F. Ferreira, Hyeokbin Kwon, Jeongwoo Yu, Leila Diniz, Henning Ulrich, Luiz R. G. Britto, Eunha Kim

**Affiliations:** a Department of Neuroscience, Korea University College of Medicine, Seoul, Republic of Korea; b Immunology Program of the Sloan Kettering Institute, Memorial Sloan Kettering Cancer Center, New York, USA; c Department of Physiology & Biophysics, Institute of Biomedical Sciences, University of São Paulo, São Paulo, Brazil; d BK21 Graduate Program, Department of Biomedical Sciences, Korea University College of Medicine, Seoul, Republic of Korea; e Department of Biochemistry, Institute of Chemistry, University of São Paulo, São Paulo, Brazil

**Keywords:** interleukin-17, neurological disorders, neurodevelopmental disorders, neurodegenerative disorders, neuropsychiatric disorders

## Abstract

In recent years, the intricate relationship between the immune system and neurological disorders has garnered significant attention. While the central nervous system had long been perceived as immunologically privileged, emerging research has revealed complex interactions among immune cells, inflammatory mediators, and neural components that collectively influence the pathogenesis and progression of various neurological disorders. This comprehensive review explores the crucial role of the cytokine interleukin-17(IL-17) in neurological diseases. It examines the complex contributions of IL-17 to both neurodevelopmental and neurodegenerative disorders, highlighting its diverse mechanisms and its impact on neuro-immune crosstalk. Moreover, we assess the evidence supporting the potential therapeutic role of modulating IL-17, considering its beneficial and detrimental effects. Through a comprehensive analysis of current research, we aim to provide insights into the modulatory role of IL-17 in neurological disorders.

## Introduction

In 1993, a gene named cytotoxic T-lymphocyte-associated protein 8 (*CTLA8*) was identified through cDNA library screening of mouse genes involved in T cell functions (Rouvier et al. 1993). CTLA8 was specifically expressed in activated T lymphocytes, and its cDNA sequence contained AU-rich repeats, commonly found in mRNAs coding for various cytokines and growth factors. Two years later, Yao and colleagues characterized the protein product of CTLA8 as a bona fide cytokine that could induce nuclear factor kappa-light-chain-enhancer of activated B cells (NF-κB) activity and interleukin (IL)-6 secretion in fibroblasts, as well as the proliferation of isolated T cells (Yao et al. 1995). The 17 kDa protein was then termed IL-17, also known as IL-17A. Later, mouse studies on experimental autoimmune encephalomyelitis (EAE) and type II collagen-induced arthritis demonstrated a specific subset of IL-17A–producing CD4^+^ T cells (Harrington et al. 2005; Langrish et al. 2005). These cells, defined as ‘T helper (Th)17’, the first-identified cellular source of IL-17A, were shown to be pro-inflammatory and essential for the pathogenesis of various autoimmune diseases, primarily mediated by IL-17A. Later, IL-17A was also identified as a crucial player in the innate-like immune response against extracellular bacterial and fungal infections, and as a key component in autoimmune diseases including psoriasis, multiple sclerosis, and ankylosing spondylitis (Acosta-Rodriguez et al. 2007; Ouyang et al. 2008; Korn et al. 2009; Zielinski et al. 2012). It is now acknowledged that Th17 cells are not the sole producers of IL-17A; other cell types including γδ T cells, natural killer (NK) cells, NKT cells, mucosal-associated invariant T (MAIT) cells, neutrophils, mast cells, and innate lymphoid cells group 3 (ILC3), have also been shown to produce IL-17A, indicating the wide-ranging influence of IL-17A signaling in the biology of immune responses (Mills 2023).

Since the initial discovery of IL-17A, five additional structurally-related cytokines have been identified: IL-17B, IL-17C, IL-17D, IL-17E (also known as IL-25), and IL-17F. These cytokines collectively form the IL-17 cytokine family (Amatya et al. 2017). The IL-17 family of cytokines functions as homodimers or heterodimers, for instance, IL-17A/F, and their corresponding receptors also present as dimers. These receptors consist of five different subunits: IL-17RA, IL-17RB, IL-17RC, IL-17RD, and IL-17RE (Li et al. 2019). IL-17RA, a common receptor subunit shared among most pairs, determines the binding ability of the cytokines. For instance, IL-17A, IL-17F, and the IL-17A/F heterodimer bind to the IL-17RA/RC pair, while IL-17E binds to the IL-17RA/RB pair (Wilson et al. 2022). Although IL-17RA is widely expressed across most tissues, variations exist in its surface expression (Ramirez-Carrozzi et al. 2011; Vidal et al. 2021). The other receptor subunits are believed to have more restricted expression patterns in specific cell types, facilitating tissue-specific responses to IL-17 cytokines (McGeachy et al. 2019).

Signaling downstream of IL-17 receptor activation begins with a specific protein domain known as similar expression to fibroblast growth factor genes (SEF) and IL-17R (SEFIR), which is conserved across all IL-17 receptor subunits (Novatchkova et al. 2003). The SEFIR domain, located within the cytoplasmic regions of IL-17 receptors, interacts with ACT1 upon ligand binding. ACT1 is an essential mediator protein integral to all recognized IL-17 signaling pathways (Chang et al. 2006; Qian et al. 2007; Liu et al. 2009). This protein features a tumor-necrosis factor receptor-associated factor (TRAF)-binding motif and lysine-63 (K63) E3 ubiquitin ligase activity, both of which are critical for TRAF activation. Consequently, various TRAFs are recruited to the SEFIR-Act1 scaffold, initiating downstream signaling events. TRAF6, in particular, is responsible for canonical IL-17 signaling that regulates gene expression at the transcriptional level (Swaidani et al. 2019). Upon binding to the IL-17 receptor complex, TRAF6 activates TAK1 (transforming growth factor-β-activated kinase 1), which then initiates the activation of nuclear factor-kappa B (NF-κB), a transcription factor that promotes the expression of various pro-inflammatory genes. Additionally, IL-17R-mediated TRAF6 activation has been reported to enhance the target genes of transcription factor activator protein-1 (AP-1) through the mitogen-activated protein kinase (MAPK) pathways, including p38, extracellular signal-regulated kinase (ERK), and c-Jun N-terminal kinase (JNK) (Cortez et al. 2007). The TRAF6-mediated pathway can be inhibited by competition from TRAF4, which shares a common TRAF-binding motif with ACT1 (Zepp et al. 2012). Beyond the SEFIR-dependent signaling, IL-17RA possesses a distinct pathway involving the C/EBPβ activation domain (CBAD), exclusively located in the cytoplasmic tail of IL-17RA (Maitra et al. 2007). This domain likely facilitates the expression of the C/EBPβ transcription factor, thereby enhancing the transcription of target genes. Additionally, it serves as a binding site for TRAF3, which can displace TRAF6, resulting in the repression of IL-17 signaling (Zhu et al. 2010).

The precise role of IL-17, particularly in modulating the gene profile, varies depending on the type of target cells, such as epithelial cells or hematopoietic cells. IL-17A is widely recognized for its ability to upregulate a diverse array of genes, including those encoding cytokines such as IL-6, granulocyte colony-stimulating factor (G-CSF), and tumor necrosis factor alpha (TNFα), chemokines such as C-X-C motif chemokine ligand 1 (CXCL1), CXCL2, and CXCL5, antimicrobial peptides such as β-defensin 2 (BD2), S100 calcium-binding protein A7 (S100A7), and lipocalin 2 (LCN2), and transcription factors such as C/EBPβ, and NF-κB inhibitor zeta (IκBζ) (Onishi and Gaffen 2010). The negative regulation of IL-17A signaling is mediated by the expression of IL-17A target genes. Notably, the expression of MCPIP1 (also known as Regnase-1), a ribonuclease, degrades mRNA for *Il6* and *Nfkbiz* (encoding IκBζ) – genes that are targets of IL-17A (Garg et al. 2015). MCPIP1 also degrades transcripts for IL-17RA and IL-17RC, thereby acting as a mechanism for receptor level suppression of IL-17A signaling cascades.

While the classical role of IL-17 involves mediating acute immune responses against extracellular bacterial and fungal infections, as well as being a key player in autoimmune diseases such as psoriasis, ankylosing spondylitis, and multiple sclerosis (MS) (Koh et al. 2024), recent findings have established IL-17 as a crucial mediator in neuro-immune interactions. IL-17 family cytokines share a cysteine knot structure with nerve growth factor (NGF) (Hymowitz et al. 2001), suggesting potential effects on the nervous system. In *Caenorhabditis elegans*, which lacks an adaptive immune system, IL-17 signaling mediates aggregation between organisms and escape response from noxious stimuli, behaviors considered to represent ancient forms of social interaction (Chen, Itakura, et al. 2017). Recent studies in mice have demonstrated the expression of IL-17 receptor subunits in different brain regions (Lee, Ishikawa, et al. 2025; Lee, Kwon, et al. 2025). Interestingly, IL-17 family cytokines modulate neuronal activity and influence various behavioral responses, including social behaviors, suggesting an evolutionarily conserved role of IL-17 signaling in the nervous system.

Given accumulating evidence for the significance of IL-17 signaling axis in the CNS, this review will explore both neuro-modulatory and pathological roles of IL-17 in neurological and neuropsychiatric disorders, with a brief overview of the current clinical progress to date and potential therapeutic strategies to target IL-17 signaling.

## Multiple sclerosis (MS)

MS is a chronic autoimmune disorder affecting the central nervous system (CNS). The destruction of the myelin sheath or oligodendrocytes by autoreactive immune cells that infiltrate the CNS leads to the formation of lesions or plaques (Constantinescu et al. 2011; Dendrou et al. 2015). This demyelination contributes to the subsequent degeneration of neuronal axons and gliosis within affected CNS regions. Clinical symptoms of MS are heterogeneous, including sensory impairments, motor disabilities, pain, and cognitive defects, which vary depending on the location of the pathological lesions. A microarray analysis of brain lesions from MS patients, using samples obtained from early post-mortem autopsies, revealed that IL-17A mRNA levels were more than two-fold higher in the lesions of MS patients compared to a control group without MS (Lock et al. 2002). IL-17A transcript levels were higher in chronic/silent lesions than in acute/active lesions, suggesting a potential association of IL-17A with MS progression. However, the precise relationship between IL-17A levels and the clinical status of the lesions appears to be complex and not merely proportional.

Experimental autoimmune encephalomyelitis (EAE) is an animal model reflecting human MS characterized by pathological lesions in the brain and spinal cord. EAE is induced in laboratory animals through immunization with myelin-related proteins or peptides, such as myelin oligodendrocyte glycoprotein (MOG), in conjunction with bacterial adjuvants, or via the adoptive transfer of myelin-reactive lymphocytes, mimicking autoimmune responses against myelin in the CNS (Miller and Karpus 2007). Production of IL-17A in both the CNS and spleen, indicating systemic involvement, was observed in mice immunized with MOG peptides to induce EAE. Furthermore, antibody-mediated neutralization of IL-17A (Hofstetter et al. 2005) and genetic deletion of IL-17A in mice significantly alleviated the clinical symptoms of EAE (Komiyama et al. 2006). These findings highlight the critical role of IL-17A in driving EAE progression. Multiple IL-17A-producing cells, including CD4^+^ T cells, CD8^+^ T cells, and astrocytes, have been identified in the active plaques of MS patients’ brains (Tzartos et al. 2008); however, CD4^+^ T cells have been identified as the primary source of IL-17A in the EAE model (Komiyama et al. 2006). Additionally, the presence of Th17 differentiation-inducing mouse intestinal commensal bacteria, segmented filamentous bacteria (SFB), influences the progression of EAE (Lee et al. 2011). This implies that immune-modulating microbiota could serve as potential therapeutic targets for neuroinflammatory diseases.

Further investigations into CNS-infiltrating IL-17A-producing cells within MS plaques have underscored the importance of Th17 cells in the pathogenesis of both EAE and MS. The prevalence of Th17 cells in the cerebrospinal fluid (CSF) was significantly higher in MS patients, particularly in those experiencing relapses compared to those in remission (Brucklacher-Waldert et al. 2009). Th17 cells in MS patients displayed increased levels of activation markers and co-stimulatory molecules compared to Th1 cells, which were previously believed to be the primary contributors to MS. In peripheral blood mononuclear cells (PBMCs), Th17 cell counts were elevated in active MS patients compared to healthy individuals or patients with inactive MS, demonstrating a positive correlation with disease activity (Durelli et al. 2009).

In addition to Th17 cells, the involvement of another effector T cell, γδ T cell, in MS pathology has been proposed. The γδ T cell, an unconventional T cell population, possesses an invariant T cell receptor (TCR) composed of γ and δ chains, as opposed to the α and β chains found in most conventional T cells (Hu et al. 2023). Unlike recognizing TCR-specific peptide antigens, γδ T cells recognize a broad array of non-peptidic antigens such as phosphoantigens, lipids, glycolipids, and heat shock proteins (HSPs), positioning themselves at the border between the innate and adaptive immune systems (Hu et al. 2023). γδ T cells also produce IL-17A, a key cytokine in MS (Korn et al. 2012). Notably, γδ T cells were found in the CSF and pathological lesions of MS patients, and they were capable of lysing human oligodendrocytes in vitro, suggesting their cytotoxic role which may contribute to the characteristics of MS pathology (Freedman et al. 1991). In the EAE model, IL-17-producing γδ T cell infiltrates were found to be significantly increased in the brain (Sutton et al. 2009). The depletion of γδ T cells with antibodies significantly ameliorated the severity of EAE, particularly in the acute phase by reducing inflammation and demyelination, whereas the chronic phase did not show significant improvement of clinical signs (Rajan et al. 1996). This suggests that γδ T cells also contribute to specific stages of MS progression, likely during the acute phase or onset of the disease.

In summary, the involvement of IL-17A-producing immune cells, particularly Th17 and γδ T cells, underscores the complexity of MS pathogenesis. Although the precise mechanisms linking IL-17A to MS progression remain to be fully elucidated, these insights offer promising directions for developing therapeutic strategies to modulate IL-17A-driven immune responses.

## Autism spectrum disorders (ASD)

**Figure 1.**
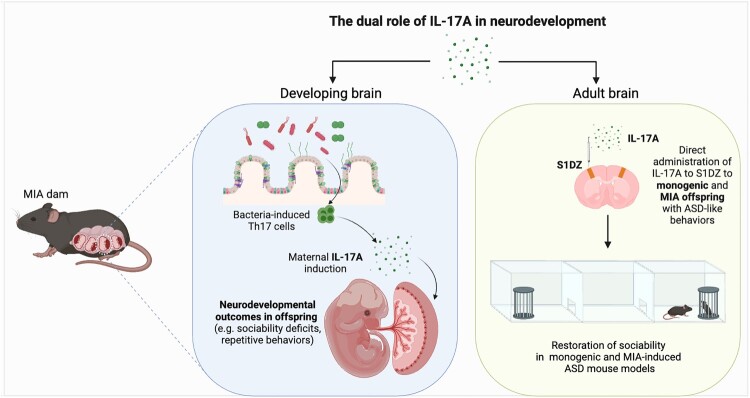
Proposed roles of IL-17A in neurodevelopmental phenotypes in ASD mouse models. The schematic illustrates how IL-17A, when maternally induced during pregnancy, impacts fetal neurodevelopment, contributing to ASD-like behavioral abnormalities in MIA offspring. Conversely, administration of IL-17A to adult mice exhibiting ASD-like behaviors can rescue these behavioral deficits.

ASD is a neurodevelopmental disorder characterized by difficulties in social interaction and stereotyped repetitive behaviors. In 2012, AL-Ayadhi and Mostafa found that children with ASD exhibited higher serum IL-17A levels than healthy controls (Al-Ayadhi and Mostafa 2012). Furthermore, they determined that the extent of behavioral abnormalities in these children was positively correlated with their serum IL-17A levels. It has also been reported that IL-17 levels are elevated in individuals with high-functioning ASD (Suzuki et al. 2011). Moreover, in animal studies using a mouse model of ASD induced by maternal immune activation (MIA) (Smith et al. 2007), induction of IL-17A in response to inflammatory challenges with synthetic double-stranded RNA, specifically polyinosinic-polycytidylic acid (poly[I:C]), has been identified as a critical factor in fetal neurodevelopment (Figure 1). Blocking the maternal induction of IL-17A effectively protected mouse offspring from neurobehavioral abnormalities (Choi et al. 2016). Maternal IL-17A has been shown to trigger an integrated stress response (ISR) in the developing brain (Kalish et al. 2021) and results in cortical hyperexcitability, particularly in the dysgranular zone of the adult primary somatosensory cortex (S1DZ) (Yim et al. 2017). Thus, IL-17A's effects are directly associated with the behavioral abnormalities observed in offspring subjected to MIA. Interestingly, the presence of SFB in the maternal gut, which induces differentiation of Th17 cells in the small intestines, has been shown to contribute to the behavioral phenotypes of MIA offspring (Kim et al. 2017; Lammert et al. 2018). These findings underscore the crucial role that IL-17 plays in neuronal development.

Recently, IL-17A has also been proposed as a potential modulator of ASD-related behaviors in mouse models. A subset of children with ASD have shown behavioral improvements during febrile episodes (Curran et al. 2007; Grzadzinski et al. 2018). Reed et al. developed a mouse model that simulates febrile episodes by administering a low dose of LPS (50 μg/kg) and demonstrated that elevated IL-17A levels in MIA offspring upon LPS exposure are critical for alleviating behavioral deficits (Reed et al. 2020). However, monogenic mouse models for ASD did not exhibit IL-17A induction by the same low dose of LPS, which may be attributed to epigenetic immune system priming in MIA offspring (Kim et al. 2022). Interestingly, direct administration of IL-17A into the brain has been found to ameliorate the behavioral abnormalities of both MIA and monogenic mouse models, highlighting the potential neuromodulatory role of IL-17A in ASD (Figure 1).

Recent studies mapped the expression of IL-17 receptor subunits across mouse brain regions, revealing distinct patterns: IL-17RA and IL-17RB are enriched in the cortex, IL-17RA and IL-17RE in the amygdala, and IL-17RC primarily in the striatum (Lee et al. 2025; Lee et al. 2025). The Predominance of IL-17RA, IL-17RB, and IL-17RC−expressing cells as neurons indicates that IL-17 cytokines, beyond IL-17A, likely modulate neuronal activity, influencing behavioral responses. These findings suggest that IL-17 cytokines play a conserved neuromodulatory role, impacting specific neuronal circuits and emphasizing the need for further research into their mechanisms beyond IL-17A.

## Alzheimer’s disease (AD)

AD is a progressive neurodegenerative disease characterized by memory loss predominantly occurring at the age of 65 or older (DeTure and Dickson 2019). The hallmark of AD is the formation of amyloid plaques – insoluble aggregates of amyloid-beta (Aβ) protein – that contribute to chronic inflammation and subsequent neurodegeneration (Kinney et al. 2018). The role of IL-17A and associated T cell populations in AD is increasingly recognized. Elevated levels of IL-17A and IL-23 – a cytokine that promotes the differentiation of Th17 cells (Iwakura and Ishigame 2006) – along with higher percentages of Th17 cells, have been found in the serum of AD patients (Chen et al. 2014). Moreover, an increased proportion of Th17 cells has been observed in the peripheral blood of patients with mild cognitive impairment due to AD pathology (Oberstein et al. 2018). In addition, T cells from AD patients exhibit increased production of IL-21, another cytokine produced by Th17 cells, and higher expression of the signature transcription factor of Th17, RORγt (Saresella et al. 2011). The administration of an IL-17A neutralizing antibody in an Aβ-injection mediated AD mouse model ameliorates cognitive impairment and neuroinflammation, underscoring the functional significance of IL-17A in AD pathogenesis (Cristiano et al. 2019). Furthermore, AD mice lacking IL-12/IL-23 exhibit a reversal in cognitive deficits (Vom Berg et al. 2012).

Stimulation of PBMCs from healthy donors with Aβ leads to increased proliferation of Th17 cells and elevated secretion of IL-17A (Yin et al. 2009). In an Aβ-injection mediated AD rat model, increased infiltration of Th17 cells and elevated IL-17A levels were observed in the brain parenchyma, hippocampus, and surrounding blood vessels (Zhang et al. 2013). Subsequent research reported that the transfer of Aβ-specific Th17 cells into amyloid precursor protein/presenilin1 (APP/PS1) transgenic AD mice worsened clinical symptoms, including memory impairment, systemic inflammation, and amyloid deposition, while reducing anti-inflammatory regulatory T cell frequencies and diminishing the immunosuppressive response (Machhi et al. 2021). Taken together, these findings suggest a role for IL-17A and Th17 cells in the pathogenesis and physiology of AD.

IL-17A – producing cells other than Th17 have also been implicated in the pathophysiology of AD. Upon activation by microglia, γδ T cells produce substantial amounts of IL-17A leading to neuronal death, which is facilitated by direct cell-cell contact with neurons (Derkow et al. 2015). Additionally, γδ T cells producing IL-17A have been reported to contribute to cognitive impairments and synaptic deficits in the triple-transgenic (3xTg) AD mouse model (Brigas et al. 2021). In the APP/PS1 and 3xTg mouse models of AD, antibody-mediated depletion of neutrophils, another immune cell type that produces IL-17A, was sufficient to ameliorate memory deficits (Zenaro et al. 2015).

**Figure 2.**
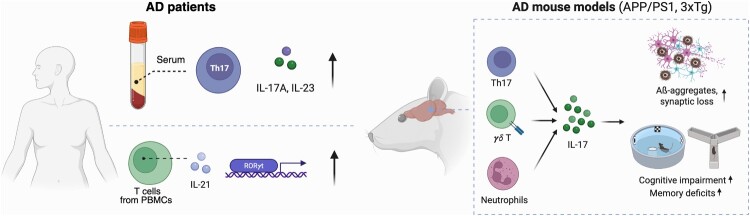
Proposed roles of IL-17A and associated immune cells in Alzheimer’s disease pathogenesis. The diagram highlights the involvement of IL-17A and IL-17 – producing cells, including Th17 cells, ɣδ T cells and neutrophils, in AD pathophysiology. Elevated levels of IL-17A and IL-23 in the serum, as well as IL-21 – producing Th17 cells along with the expression of their signature transcription factor, RORɣt, in PBMCs, have been observed in AD patients. Mouse models of AD have shown that brain IL-17A production by immune cells leads to neuronal death, synaptic loss, and an exacerbation of AD-like behaviors.

To summarize, recent evidence highlights the critical role of IL-17A and IL-17A – producing cells in the pathogenesis of Alzheimer's Disease (AD) (Figure 2). Future studies should seek to clarify how IL-17A contributes to neurodegeneration and to explore interventions that modulate this immune response. Advancing our understanding of these dynamics could lead to innovative approaches for reducing the neuroinflammatory load in AD, thereby enhancing patient outcomes.

## Parkinson’s disease (PD)

PD is a neurodegenerative disease with a high prevalence in individuals over the age of 65. In 2019, the global prevalence of PD had increased by 155.50% compared to 1990. In line with this, the years living with disability – an index used to evaluate health loss and disease burden – rose substantially by 154.73% since 1990 (Ou et al. 2021). Due to the lack of effective disease-modifying drugs (Hauser 2009), recent research has been directed toward identifying alternative molecular targets and pathways to manage PD pathology. These include calcium homeostasis, mitochondria, α-synuclein, oxidative stress, neurotrophic factors, and neuroinflammation. For instance, antagonists to purinergic P2X7 and P2Y6 receptors (Ferrazoli et al. 2017; Oliveira-Giacomelli et al. 2019; Glaser et al. 2020; Silva et al. 2023), TRP channel blockers (Dati et al. 2017; Ferreira et al. 2022; Joe Steinman et al. 2022; Ferreira and Britto 2023; Ferreira, Ulrich, Feng, et al. 2024; Ferreira, Ulrich, Mori, et al. 2024), and strategies targeting the depletion of α-synuclein (Kim et al. 2019) or microglia (Pereira et al. 2023) have all proven effective in improving both the motor and non-motor symptoms of PD in rodent models.

The involvement of IL-17A and IL-17A – producing cells in the pathophysiology of PD has been widely reported, but these findings continue to be debated. Research comparing untreated PD patients with healthy individuals demonstrated a marked decrease in peripheral plasma IL-17A levels in PD (Alvarez-Luquin et al. 2019) patients. Moreover, Green et al. reported a correlation between IL-17A levels and non-motor symptoms of PD (Green et al. 2019). This study also revealed that female PD patients exhibited significantly higher plasma IL-17A levels than male patients, with IL-17A levels positively correlating with anxiety but inversely with cognitive deficits. These findings indicate that plasma IL-17A levels can be influenced by disease stage and gender.

Postmortem brain tissues from PD patients showed significant accumulation of CD3^+^ T cells, but not of B or NK cells (Brochard et al. 2009; Sommer et al. 2019). Newly diagnosed, medication-free PD patients were found to have elevated numbers of IL-17A^+^ Th17 cells in peripheral blood, underscoring the pathogenic role of Th17 cells in PD (Chen, Liu, et al. 2017; Sommer et al. 2019). However, another study reported a reduced number of circulating CD4^+^ T cells, including Th17 cells, in both untreated and medication-treated PD patients (Kustrimovic et al. 2018). These variations in Th17 cell numbers could be attributed to the fact that measurements were conducted in peripheral blood, not in the brain parenchyma of PD patients.

Molecular mechanisms of IL-17A actions on neurons in PD have been explored in recent decades. Although further investigation is required, dorsal root ganglion neurons and mouse hippocampal neurons are reported to express IL-17RA (Segond von Banchet et al. 2013; Luo et al. 2019; Di Filippo et al. 2021). IL-17RA expression in dopaminergic neurons has also been confirmed in human induced pluripotent stem cell (hiPSC)-derived midbrain neurons from PD patients (Sommer et al. 2018). Sommer et al. reported significantly upregulated IL-17R expression in hiPSC-derived neurons from PD patients, leading to increased susceptibility to IL-17A – induced neuronal death, potentially via NF-κB signaling activation. Liu et al. suggested that IL-17A from Th17 cells exacerbated dopaminergic neuronal death through microglia and TNFα-mediated cell death (Liu et al. 2019). In mouse models of PD, Th17 cells specifically infiltrated dopaminergic neuron-rich brain regions. Additionally, neutralizing antibodies blocking adhesion molecules necessary for Th17 cell entry into the brain parenchyma, such as leukocyte function-associated antigen (LFA)−1 or intercellular adhesion molecule (ICAM)−1, abolished Th17-mediated dopaminergic neuronal cell death (Liu et al. 2017).

α-synuclein is being investigated as a potential autoantigen for T cell-mediated immune responses in PD. PBMCs from PD patients were activated and produced cytokines in response to α-synuclein peptide epitopes, with the most responsive cells being IFNγ-producing CD4^+^ T cells (Sulzer et al. 2017). While this study did not directly implicate Th17 cells, it underscored their potential role in the autoimmune responses affecting PD progression, similar to observations in MS. Additional evidence suggests that the transfer of α-synuclein-immunized Th17 cells exacerbated the pathology in a 1-methyl-4-phenyl-1,2,3,6-tetrahydropyridine-induced model of PD, further implicating Th17 cells in PD pathology (Reynolds et al. 2010).

**Figure 3.**
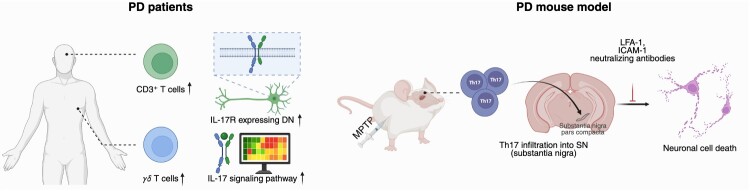
Potential roles of IL-17 and IL-17 – producing immune cells in PD. The schematic highlights the involvement of IL-17A and IL-17 – producing cells, including Th17 and ɣδ T cells in PD. Elevated Th17 and ɣδ T cells in PBMC and CSF were observed in PD patients. IL-17RA expression in dopaminergic neurons and related IL-17 downstream genes were upregulated in PD patients. In a PD mouse model, neutralization of IL-17A or blockade of Th17 infiltration ameliorated neuronal death.

In addition to the importance of Th17 cells, the role of γδ T cells in PD has also been elucidated. Patients with PD exhibited significantly higher levels of γδ T cells in both peripheral blood and CSF compared to those with other neurological disorders such as polyneuropathy, cerebrovascular disease, and epilepsy (Fiszer et al. 1994). Recent research provided further evidence of the involvement of both Th17 and γδ T cells by analyzing PBMCs from PD patients (Diener et al. 2023). Transcriptomic analyses of peripheral T cells from five representative cases of PD revealed an increase in the KEGG (Kyoto Encyclopedia of Genes and Genomes) category ‘IL-17 signaling pathway’ among genes expressed differently between PD patients and healthy subjects. This category includes not only key transcription factors for Th17 differentiation such as RORγ and basic leucin zipper ATF-like transcription factor (*BATF*), but also factors related to IL-17 signaling, including *IL17D, IL17F, IL6,* prostaglandin-endoperoxide synthase 2 (*PTGS2*)*,* chemokine ligand 20 (*CCL20*). Subtype profiling of peripheral T cells highlighted these findings, showing a significantly elevated prevalence of both Th17 and γδ T cells, particularly a subtype producing IL-17A. Collectively, these findings emphasize the critical role of IL-17 and its downstream effects in PD pathogenesis and call for a detailed investigation into their mechanisms in regulating disease progression (Figure 3).

## Depression

Major depressive disorder (MDD) is one of the most common neuropsychiatric diseases of childhood and adolescence (Lim et al. 2018; Mullen 2018). Risk factors for MDD include physical and psychological stresses, which can cause long-term and recurrent depressive episodes. Additionally, there is a known association between abnormal and chronic inflammatory responses and the severity of depressive symptoms (Han and Yu 2014). Recent studies have proposed potential associations between IL-17 and the development, severity, and treatment resistance of depressive disorders (Waisman et al. 2015; Davami et al. 2016; Nothdurfter et al. 2021); however, these findings continue to be the subject of debate (Liu et al. 2012; Kim et al. 2013).

**Figure 4.**
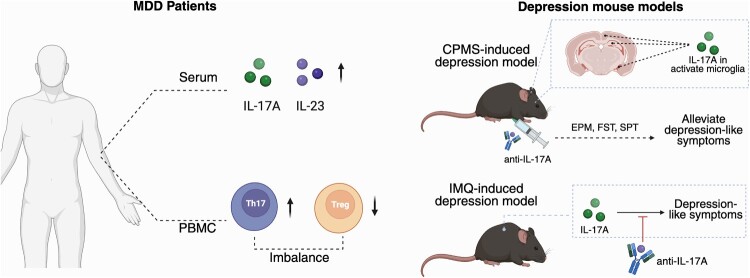
Potential roles of IL-17 and IL-17 – producing immune cells in MDD. The schematic highlights the involvement of IL-17A and IL-17 – producing cells in MDD and depression mouse models. Elevated Th17-related cytokines have been observed in the serum of MDD patients, and an imbalance between Th17 and Treg cells has been reported. Neutralization of IL-17A in both CPMS- and IMQ-induced depression models alleviated depression-like behaviors in mice.

In a study involving 190 patients diagnosed with depression, excluding those with coexisting inflammatory diseases, compared to 100 healthy individuals, elevated serum levels of IL-17 and IL-23 along with decreased levels of IL-21 and IL-35, a cytokine that counterbalances IL-17 (Galecka et al. 2021), were observed. Other studies have reported an elevation in the levels of Th17 cells in the peripheral blood (Chen et al. 2011) and serum IL-17A in patients with depression compared to healthy controls (Davami et al. 2016) (Figure 4).

The potential role of IL-17 in depression is further substantiated by the antidepressant effects of ketamine. Notably, elevated levels of circulating IL-17 have been observed in individuals with depression, alongside a reduction in Th17 cells following ketamine treatment (Cui et al. 2021). Mao et al. established a correlation between serum IL-17 concentrations and the severity of first-episode depressive disorders (Mao et al. 2022). Furthermore, an imbalanced ratio between Th17 cells and anti-inflammatory regulatory T cells (Treg) has been reported in MDD (Chen et al. 2011).

In a mouse model of depression induced by cumulative mild stress (CPMS), IL-17 was found to activate microglia in the hippocampus, amygdala, and prefrontal cortex. Th17 cells were also detected in the brains of CPMS mice, and treatment with an anti-IL-17 antibody alleviated depression-like symptom (Kim et al. 2021). Similarly, in a mouse model of psoriasis using imiquimod (IMQ), depression-like symptoms were observed alongside IL-17A induction, and treatment with an anti-IL-17A antibody led to a reduction in IMQ-induced depression-like behaviors (Nadeem et al. 2017). A phase 3 clinical trial for treating psoriasis with the anti-IL-17 receptor monoclonal antibody, Brodalumab, also supported prior in vivo findings, showing amelioration of depressive symptoms – measured by the Hospital Anxiety and Depression Scale – alongside improvements in psoriasis symptoms (Papp et al. 2016) (Figure 4).

While these findings underscore the potential involvement of IL-17 in depression, other research indicates that its impact may be modulated by variables such as age and epigenetic factors. For example, in late-life depression (LLD), studies showed that IL-17 plasma levels were not elevated in elderly patients, as evidenced by research involving 74 LLD patients and 55 non-depressed individuals (Saraykar et al. 2018). However, IL-17A levels correlated with age, consistent with findings by Spanemberg et al. (Spanemberg et al. 2014). These observations prompt important questions regarding the interactions of IL-17 in aging and depressive behaviors. Consequently, further investigation is required to clarify the mechanisms through which IL-17 contributes to depression.

## Future aspects: exploring the IL-17 pathway in neurological disorders and the gut microbiome

IL-17A is recognized as a significant therapeutic target in various inflammatory disorders. Monoclonal antibodies such as Bimekizumab, Secukinumab, Ixekizumab, and Brodalumab, which inhibit IL-17A or IL-17RA, have received FDA approval for managing psoriasis and psoriatic arthritis (Simopoulou et al. 2023). In a Phase 2 clinical trial, Secukinumab demonstrated encouraging outcomes among patients with relapsing-remitting MS (Havrdova et al. 2016). Neutralizing IL-17A in an AD mouse model mitigated cognitive decline (Cristiano et al. 2019). Moreover, inhibiting IL-17A in a rat PD model lessened motor deficit, provoked effects potentially associated with alterations in the microglial phenotype and function (Liu et al. 2019). These findings indicate that neuroprotection may be achieved by disrupting the interaction between IL-17A and its receptor, highlighting its potential as a therapeutic target.

Differentiation of immune cells producing IL-17A, including γδ T cells, Th17, and mucosal associated invariant T cells (MAIT), is known to be regulated by the microbiota (Tanoue et al. 2010; Legoux et al. 2020; Papotto et al. 2021). SFB, for example, is a well-defined inducer of Th17 in the small intestines of mice (Ivanov et al. 2009). Subsequent investigations have also identified human commensal microbiota capable of inducing Th17 in the human intestines (Atarashi et al. 2015; Tan et al. 2016; Geva-Zatorsky et al. 2017). As previously described, the presence of SFB in pregnant dams contributes to the induction of IL-17A upon immune activation and influences offspring neurodevelopment. γδ T cells, a major immune cell population in the meninges and prolific producers of IL-17A, depend on gut microbiota for their presence in the meninges (Alves de Lima et al. 2020). Meningeal γδ T cells, alongside MAIT cells, are known to influence brain function and behavior in mice (Zhang et al. 2022).

Alterations in intestinal microbial composition have been observed in various neurological and neuropsychiatric disorders, including ASD, depression, AD, and PD (Scheperjans et al. 2015; Vogt et al. 2017; Sharon et al. 2019; Strandwitz et al. 2019). Emerging evidence suggests the modulation of the IL-17 pathway by intestinal microbiota as a possible mechanistic link between microbial dysbiosis and the pathophysiology of these neurological disorders. For instance, recent research has shown a pivotal role of the gut microbiome in modulating IL-17A levels and impacting AD pathology (Hao et al. 2024). Depletion of gut bacteria in APP/PS1 AD mouse models significantly reduced Th17 cells in both the spleen and gut, leading to decreased neuroinflammatory activation and reduced Aβ deposition in the brain. Moreover, antibiotic-induced gut microbiome depletion increased the expression of key Aβ clearance transporters, such as LRP1 and ABCB1, at the blood-brain barrier, a process reversibly blocked in IL-17A KO mice. Concentrations of short-chain fatty acid (SCFA) metabolites were significantly lower in PD patient feces (Unger et al. 2016), significant because SCFAs, produced by specific gut microbes, have been shown to modulate intestinal motility via the enteric nervous system and promote IL-17 expression in γδ T cells. The impact of gut microbiota on PD has been experimentally validated by transplanting microbiota from PD patients into mice. In a mouse model overexpressing α-synuclein to mimic PD synucleinopathies, the presence of gut microbes exacerbated motor deficits and intestinal dysfunctions characteristic of PD (Sampson et al. 2016).

Taken together, modulating the gut microbiota, which may influence both gut-residing and CNS-associated immune cells that produce IL-17A, represents a promising therapeutic strategy for neurological disorders characterized by chronic inflammation. Given the potential link between microbial dysbiosis and IL-17A−mediated neuroinflammation in various neurological disorders, interventions targeting gut microbiota could offer new avenues for slowing disease progression. By integrating microbiome modulation with immune-targeted approaches, more effective treatments may be developed that address both peripheral and central immune mechanisms contributing to neurological and neuropsychiatric disorders.

## Conclusion

Historically, the CNS was regarded as an immune-privileged site, protected from peripheral immune surveillance by specialized barriers such as blood-brain barrier (BBB). However, recent investigations have prompted a re-evaluation of the concept of the brain as an immunologically privileged organ (Louveau et al. 2015; Smyth et al. 2024). These studies have provided evidence supporting the hypothesis that immune pathways have been co-opted to exert a direct influence on neural circuits, affecting not only those associated with pathological neuroinflammation but also those involved in the regulation of animal behaviors and mood.

Since its initial discovery, IL-17A has been recognized as a critical mediator with a wide-ranging role in peripheral immune responses. IL-17A is also implicated as a key contributor to CNS autoimmune disorders such as MS and neurodegenerative diseases including AD and PD. Moreover, its role has recently been shown to extend beyond traditional immunological paradigms, influencing neuronal activity and animal behaviors (Alves de Lima et al. 2020; Reed et al. 2020; Lee, Ishikawa, et al. 2025; Lee, Kwon, et al. 2025). However, our current understanding of the action of IL-17 in the CNS remains limited, and significant gaps exist in delineating how IL-17–mediated pathways in the CNS may differ from classical immune cell pathways. Therefore, future studies are needed to investigate the downstream IL-17 receptor signaling in CNS compared to classical immune pathways.

Immune system function fundamentally relies on maintaining balance. While certain levels of cytokine activity, including IL-17A, are essential for host protection and tissue homeostasis, excessive or dysregulated cytokine signaling can lead to pathological inflammation. In particular, IL-17 receptor signaling downstream of ACT1-TRAF6 induces the expression of negative regulators to constrain overactivation (McGeachy et al. 2019). However, under pathological conditions such as autoimmune diseases, the synergy between IL-17A and other pro-inflammatory cytokines – including TNF, IL-1, and IFN-γ – amplifies inflammatory responses, ultimately leading to tissue damage (Griffin et al. 2012). These reports suggest that IL-17A alone is often insufficient to trigger pathology without such synergistic inflammatory environments. Therefore, in severe neuroinflammatory states, the integrity of anatomical barriers such as BBB can be compromised, allowing peripheral immune cells, including T cells, to infiltrate the CNS (Nichols et al. 2009; Chen et al. 2024). This transition from a cytokine-mediated modulation to direct immune cell infiltration marks a critical shift in the role of IL-17A from maintaining homeostasis to contributing to pathology. Thus, IL-17A functions differently across disease stages it supports neuroimmune balance and behavior modulation, whereas in severe pathological states, it acts synergistically with other inflammatory mediators to exacerbate CNS inflammation and tissue injury.

In addition to canonical IL-17A producers such as T cells, glial cells within the CNS – particularly astrocytes and microglia – have emerged as both targets and active participants of IL-17A signaling. Although microglia and astrocytes express IL-17RA and IL-17RC, stimulation with IL-17A alone appears insufficient to induce cytokine or chemokine expression from these cells (Nichols et al. 2009). However, IL-17A has been shown to potentiate TNF-induced responses suggesting that co-stimulatory inflammatory signals are required to elicit pathogenic effects of IL-17A within the CNS. Astrocytes itself can also produce IL-17A, thereby contributing directly to local neuroinflammation, particularly in neuroinflammatory autoimmune disease such as MS (Tzartos et al. 2008). Microglia, upon IL-17A stimulation, adopt pro-inflammatory phenotypes that promote neuronal injury in models of AD (Liu et al. 2019) and depression (Kim et al. 2021). These findings highlight that IL-17A does not act solely via infiltrating periphery immune cells but also orchestrates neuroimmune interactions among resident glial cells in CNS.

In summary, IL-17A has evolved from being recognized solely as a peripheral pro-inflammatory cytokine to an essential modulator of CNS function, contributing to both physiological neuromodulation and pathological neuroinflammation. Its involvement spans a wide range of neurological and neuropsychiatric disorders, including MS, ASD, AD, PD, and depression, through complex interactions with neurons, glial cells, and immune cells. However, the molecular level of understanding by which IL-17A signaling operates differently in CNS compared to classical immune system remain to be understood. Future research for dissecting CNS-specific IL-17 receptor signaling pathways, clarify the context-dependent roles of IL-17A across health and disease, and explore how gut microbiota-mediated modulation of IL-17A impacts brain function are expected. A deeper understanding of IL-17A’s dual roles – balancing neuroimmune homeostasis versus driving neuroinflammation – may open new therapeutic avenues to selectively target pathological IL-17A signaling while preserving its essential physiological functions in the CNS.
